# Highly Selective and Sensitive Fluorescent Probe for Copper (II) Ions Based on Coumarin Derivative with Aggregation-Induced Emission

**DOI:** 10.3390/s26072087

**Published:** 2026-03-27

**Authors:** Jie Liu, Peng Chen, Guoyu Guo, Xinbo Gao, Yaozu Xie, Zikang Li, Zhen Zhang, Shuisheng Chen

**Affiliations:** Anhui Provincial Key Laboratory of Green Carbon Chemistry, School of Chemistry and Chemical Engineering, Fuyang Normal University, Fuyang 236037, China

**Keywords:** Schiff base derivatives, aggregation-induced emission, Cu^2+^ recognition and analysis, selective fluorescence turn-off, environmental monitoring and protection

## Abstract

Excessive accumulation of copper ions (Cu^2+^) in the environment and biological systems poses severe risks to ecological balance and human health, necessitating accurate detection and monitoring of Cu^2+^. Schiff base derivatives with favorable optical properties provide an efficient strategy for copper ion recognition. In this paper, fluorescent probe **L** (5-methyl-2-hydroxybenzaldehyde-(7-diethylaminocoumarin-3-formyl) hydrazone) was synthesized through a three-step reaction using 4-diethylaminosalicylaldehyde and diethyl malonate as starting materials. The structure of probe **L** was confirmed by melting point analysis, infrared spectroscopy, and nuclear magnetic resonance. Single-crystal X-ray analysis revealed that probe **L** crystallized into a triclinic lattice with space group $P_{1}^{-}$. Optical investigations, including UV–Vis spectroscopy, fluorescence spectroscopy, and aggregation-induced emission studies, demonstrated highly sensitive and selective fluorescence “turn-off” behavior of probe **L** towards Cu^2+^ ions in DMSO, with negligible interference from other metal ions. Job’s plot and crystallographic analysis revealed a 1:1 binding stoichiometry between probe **L** and Cu^2+^, forming the complex [Cu(L)]. Fluorescence titration experiments revealed a binding constant (*K_b_*) of 5.2 × 10^6^ L/mol and a detection limit of 7.8 × 10^−7^ mol/L, indicating excellent sensitivity. These results suggest that probe **L** has considerable promise for Cu^2+^ detection in aqueous environments, with potential applications in environmental monitoring and public health protection.

## 1. Introduction

Monitoring heavy metal ion levels has become a global research priority due to their harmful impact on the environment and human health [1]. Among them, copper ions (Cu^2+^) have particular significance. As the third most abundant metal ion in the human body, Cu^2+^ is an essential trace element for life, participating in the formation of various enzyme complexes and playing a crucial role in maintaining the normal function of the central nervous system [2]. The intracellular concentration of Cu^2+^ is tightly regulated. Disruption of copper homeostasis can lead to a range of diseases, including liver cirrhosis, Alzheimer’s disease (AD), Parkinson’s disease (PD), and Wilson’s disease (WD) [3,4,5]. Excessive copper ions also exert irreversible effects on plants. For example, they interfere with photosynthesis in phytoplankton, thereby affecting aquatic ecosystems [6]. Given the above concerns, the development of Cu^2+^ detection methods with simple synthesis procedures, excellent selectivity, high sensitivity, strong anti-interference capability, and good stability is of significant application value.

Currently, the primary detection methods for Cu^2+^ mainly include atomic absorption spectrometry, cyclic voltammetry, potentiometry, inductively coupled plasma emission spectrometry, and others [7,8,9,10]. However, these conventional approaches are often limited by prolonged testing durations and high operational costs, which significantly restrict their widespread application in scenarios requiring rapid and cost-effective analysis. In contrast, fluorescent probe-based detection methods exhibit distinct advantages, such as low cost, high selectivity and sensitivity, low detection limits, rapid response, convenient visualization, and the ability to conduct in situ quantitative detection and analysis.

To date, numerous fluorescent probe-based detection methods have been developed for Cu^2+^, using compounds such as isophorones [11], rhodamines [12], pyridines [13], dansylamides [14], naphthalimides [15], triphenylamine [16], fluorescein [17], and coumarins [18]. Among these, coumarin structures have garnered extensive attention due to their inherent advantages, including high fluorescence quantum yield, large Stokes shift, excellent photostability, visible excitation and emission wavelengths, and low toxicity [19,20,21]. Specifically, when an electron-withdrawing group (such as formylhydrazine) is introduced at the 3-position and an electron-donating group (e.g., diethylamino group) is at the 7-position, the coumarin π-electron cloud undergoes intramolecular charge transfer (ICT) driven by the push–pull effect of these two groups. This modification endows the resulting compound with exceptional fluorescent properties. In the presence of metal ions, the phenolic hydroxyl group tends to deprotonate, and the chemosensor acts as a negative chelator to bind to metal ions. At the same time, the lone electron pairs on the imino group also bind to the metal ions, resulting in red shifts in both excitation and emission bands.

Signal transduction in such systems generally occurs via chelation-enhanced fluorescence quenching (CHEQ), which arises from the formation of a centrosymmetric dinuclear structure upon chelation of Cu^2+^. Schiff base derivatives derived from coumarins exhibit advantageous characteristics, such as fewer synthetic steps, high yield, low toxicity, favorable photostability, and high sensitivity [22,23]. These practical benefits highlight the potential of Schiff base reactions as valuable and efficient strategies for ion-tracing synthesis.

**Figure 1:**
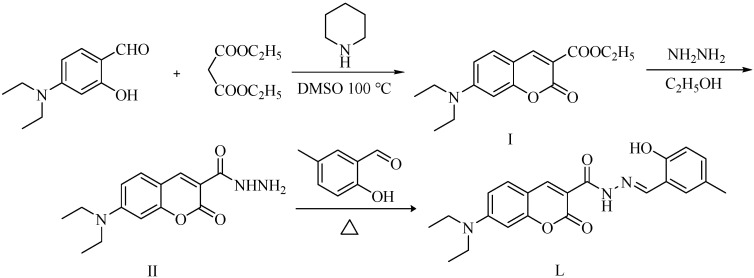
Synthetic route of probe **L**.

In this study, a novel fluorescence probe was developed for the selective detection of copper ions. The starting materials of 4-diethylaminosalicylaldehyde and diethyl malonate first underwent the Knoevenagel condensation reaction under weakly alkaline conditions to yield intermediate I, which was then subjected to a hydrazinolysis reaction with hydrazine hydrate to afford the key intermediate II. Subsequently, intermediate II reacted with 5-methylsalicylaldehyde through nucleophilic addition to synthesize the acylhydrazone-based Schiff base probe **L** [5-methyl-2-hydroxybenzaldehyde-(7-diethylaminocoumarin-3-formyl)hydrazone] (as shown in Figure 1). The structure of probe **L** was characterized by melting point determination, infrared spectroscopy, nuclear magnetic resonance spectroscopy, and single-crystal X-ray diffraction. Furthermore, based on the investigation of the aggregation-induced emission (AIE) property of probe **L**, its application in the recognition of Cu^2+^ was explored.

## 2. Materials and Methods

### 2.1. Reagents and Apparatus

All reagents used for the synthesis of probe **L** were purchased from Energy Chemical Reagent (Yousheng Biotechnology Co., Ltd., Hefei, Anhui, China) and used directly without further purification.

FT-IR spectra were recorded on a WQF-51 Fourier Transform Infrared Spectrometer (Rayleigh, Beijing, China). ^1^H and ^13^C NMR spectra were acquired on an Ascend 400 spectrometer (Bruker, Bruk, Switzerland) with TMS as the internal standard and CDCl_3_ as the solvent. UV–Vis absorption and fluorescence emission spectra were recorded on a TU-1901 Double-Beam UV-Vis Spectrophotometer (Puxi, Beijing, China) and F97Pro Fluorescence Spectrophotometer (Lengguang, Shanghai, China). The melting point was determined using X-5A Micro Melting Point Apparatus (Gongyi, Henan, China). Single-crystal X-ray diffraction data were collected on an XtaLAB Synergy CCD diffractometer (Rigaku, Tokyo, Japan).

### 2.2. Synthesis of Intermediates ***I*** and ***II*** and Probe ***L***

#### 2.2.1. Synthesis of Intermediate **I** (Ethyl 7-Diethylaminocoumarin-3-carboxylate)

4-Diethylaminosalicylaldehyde (6 g; 31.05 mmol) was placed in a clean 50 mL round-bottom flask followed by the addition of 35 mL DMSO. The mixture was stirred at room temperature, and then diethyl malonate (7.5 mL; 49.40 mmol) was added. Subsequently, piperidine (15 drops) was carefully added into the reaction mixture dropwise, and the mixture was refluxed at 100 °C for 3 h. Then, 30 mL of 0 °C ice water was added to the reaction mixture, and it was kept in a 4 °C constant-temperature refrigerator for 30 min. The resulting crystalline solid was filtered and washed with 15% ethanol. After recrystallization from a solution of absolute ethanol:cyclohexane = 1:10, product **I** was obtained (7.76 g; 86.5%): m.p. 81.9–82.3 °C; ^1^H-NMR (400 MHz; Chloroform-*d*)—*δ:* 8.44 (s, 1H), 7.37 (d, *J* = 8.9 Hz, 1H), 6.64 (dt, *J* = 8.9, 2.5 Hz, 1H), 6.49 (d, *J* = 2.3 Hz, 1H), 4.38 (q, *J* = 7.1 Hz, 2H), 3.45 (q, *J* = 7.1 Hz, 4H), 1.39 (t, *J* = 7.1 Hz, 3H), and 1.24 (t, *J* = 7.1 Hz, 6H).

#### 2.2.2. Synthesis of Intermediate **II** (7-Diethylaminocoumarin-3-formylhydrazine)

A mixture of ethyl 7-diethylaminocoumarin-3-carboxylate (2.89 g; 10 mmol) and hydrazine hydrate (80%; 3 mL; 60 mmol) in 40 mL of ethanol was stirred at room temperature for 0.5 h. The resulting crystalline solid was filtered and washed with 50% ethanol. After recrystallization from a solution of absolute dichloromethane:cyclohexane = 1:1, product **II** was obtained (2.48 g; 90.2%): m.p. 171.7–173.1 °C; ^1^H-NMR (400 MHz; DMSO-*d*_6_)—*δ:* 9.45 (s, 1H), 8.65 (s, 1H), 7.70 (d, *J* = 9.0 Hz, 1H), 6.81 (dd, *J* = 9.0, 2.5 Hz, 1H), 6.61 (d, *J* = 2.4 Hz, 1H), 4.62 (s, 2H), 3.48 (q, *J* = 7.0 Hz, 4H), and 1.14 (t, *J* = 7.0 Hz, 6H).

#### 2.2.3. Synthesis of Probe **L** [5-Methyl-2-hydroxybenzaldehyde-(7-diethylaminocoumarin-3-formyl) hydrazone]

5-methylsalicylaldehyde (0.0681 g; 0.5 mmol) was added to a solution of 7-diethylaminocoumarin-3-formylhydrazine (0.138 g; 0.5 mmol) in ethanol (50 mL). Following the addition of 2 drops of glacial acetic acid, the mixture was refluxed at 80 °C for 6 h. After the reaction mixture was allowed to stand overnight at room temperature, it was subjected to suction filtration, rinsed with absolute ethanol, and dried to afford the target product **L** (0.1318 g; 67%): m.p. 241.1–242.6 °C; IR: 1269.02 (C-N), 1695.84 (C=O), 3447.48 (N-H), 1194.36 (N-N), and 1133.73 (C-O-C); ^1^H-NMR (400 MHz; DMSO-*d*_6_)—*δ:* 11.79 (s, 1H), 11.02 (s, 1H), 8.75 (s, 1H), 8.61 (s, 1H), 7.74 (d, *J* = 9.04 Hz, 1H), 7.30 (s, 1H), 7.12 (dd, *J* = 8.32 Hz, 1.96 Hz, 1H), 6.84 (t, *J* = 7.68 Hz, 2H), 6.68 (s, 1H), 3.51 (q, *J* = 6.92 Hz, 4H), 2.25 (s, 3H), and 1.15 (t, *J* = 6.96 Hz, 6H); ^13^C NMR (101 MHz; DMSO-*d*6)—*δ:* 161.19, 158.80, 157.32, 155.21, 152.73, 148.76, 148.45, 132.07, 131.77, 129.40, 127.76, 118.11, 116.16, 110.28, 108.04, 107.74, 95.84, 44.31, 19.85, and 12.22.

### 2.3. X-Ray Crystallography

A single crystal of appropriate size was selected for single-crystal X-ray diffraction analysis, and data collection was performed on a RigakuSmartlab X-ray diffractometer equipped with a graphite monochromator, using MoKα radiation (λ = 0.71069 Å) and ω/2θ scanning mode. The crystal structures were solved by using structure solution program SHELXT (Version: SHELXT 2018/2) with the Intrinsic Phasing method with F^2^ defined via the full-matrix least-squares technique in OLEX2 software. The coordinates of hydrogen atoms were obtained through difference Fourier synthesis while those of non-hydrogen atoms were determined by the direct method; hydrogen atoms were refined with isotropic thermal parameters, whereas all other atoms were subjected to refinement with anisotropic thermal parameters.

### 2.4. Optical Property Testing of Fluorescent Probe ***L***

A stock solution of **L** (3.3 × 10^−4^ M) was prepared in DMF. Test solutions were prepared by placing 50 μL of the probe stock solution into a 5 mL volumetric flask, followed by the addition of 4.95 mL of seven different solvents (benzene, dichloromethane, ethyl acetate, tetrahydrofuran, ethanol, acetonitrile, and DMF). The ultraviolet–visible absorption spectra and fluorescence emission spectra of each of these diluted solutions were measured.

### 2.5. Cu^2+^ Recognition Ability of Fluorescent Probe ***L***

#### 2.5.1. Selectivity Experiment

A stock solution of **L** (3.3 × 10^−4^ M) was prepared in DMF, and stock solutions of various nitrate salts of cations (3.3 × 10^−4^ M; Ag^+^, Al^3+^, Ca^2+^, Cd^2+^, Co^2+^, Li^+^, Mg^2+^, Na^+^, Ni^2+^, Cu^2+^, Sr^2+^, Zn^2+^, K^+^, Hg^2+^, Pb^2+^, Bi^3+^, Cr^3+^, Mn^2+^, Ba^2+^, and Fe^3+^) were prepared in ethanol. These cations were selected as they are known interferents of copper ions.

Test solutions were prepared by placing 50 μL of the probe stock solution into twenty 5 mL volumetric flasks containing 4.80 mL DMSO and then adding an appropriate aliquot of each metal stock solution with a micropipette. Their fluorescence emission spectra were determined.

#### 2.5.2. Fluorescence Titration Experiment

Test solutions were prepared by placing 200 μL of the probe stock solution into sixteen 5 mL volumetric flasks. Then, 0 μL, 20 μL, 40 μL, 60 μL, …, and 300 μL of Cu(NO_3_)_2_ ethanol solution (3.3 × 10^−4^ mol/L) were added to each volumetric flask, respectively. Each mixture was diluted to the calibration mark with DMSO, and its UV–Vis absorption spectra and fluorescence emission spectra were measured.

#### 2.5.3. Anti-Interference Experiment

Test solutions were prepared by placing 50 μL of the probe stock solution and 150 μL of Cu^2+^ ethanol solution into twenty 5 mL volumetric flasks. Subsequently, 150 μL of each of the stock solutions of various cations (3.3 × 10^−4^ mol/L) was added separately to the corresponding volumetric flasks. Each mixture was then diluted to the calibration mark with DMSO, and the fluorescence emission spectra were measured.

#### 2.5.4. Complexation Ratio Experiment

In ten 5 mL volumetric flasks, the total volume of the DMF solution of probe **L** (3.3 × 10^−4^ mol/L) and the ethanol solution of Cu(NO_3_)_2_ (3.3 × 10^−4^ mol/L) was kept constant at 200 μL, and ten different molar ratios of probe **L** to Cu(NO_3_)_2_, namely 200:0, 180:20, 160:40, 140:60, …, and 20:180, were prepared. Each mixture was then diluted to the calibration mark with DMSO, and the fluorescence emission spectra were measured.

## 3. Results and Discussion

### 3.1. Synthesis

A simple three-step reaction protocol was employed to synthesize the target probe **L**. First, 4-diethylaminosalicylaldehyde and diethyl malonate were employed as starting materials to yield intermediate I via the Knoevenagel reaction. Then, needle-like crystals of intermediate I were obtained by slow evaporation of its saturated anhydrous ethanol–cyclohexane (1:10, *v*/*v*) solution. Intermediate I crystallized into a monoclinic lattice with space group *P*2_1_/c (Figure S1 and Table S1). The hydrazinolysis reaction of intermediate I with hydrazine hydrate yielded the key intermediate II. Needle-like crystals of intermediate II were obtained by slow evaporation of its saturated dichloromethane–cyclohexane (1:1, *v*/*v*) solution. Intermediate II crystallized into a triclinic lattice with space group $P_{1}^{-}$ (Figure S2 and Table S1).

**Figure 2:**
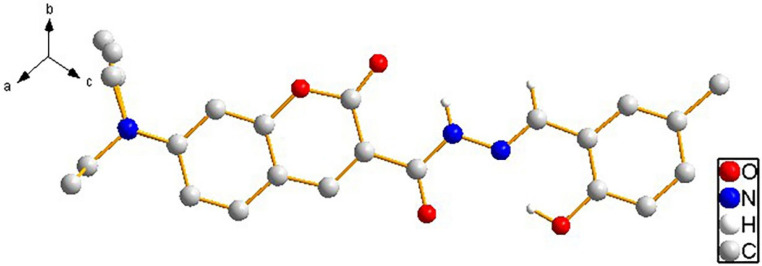
Molecular structure of compound **L**.

**Figure 3:**
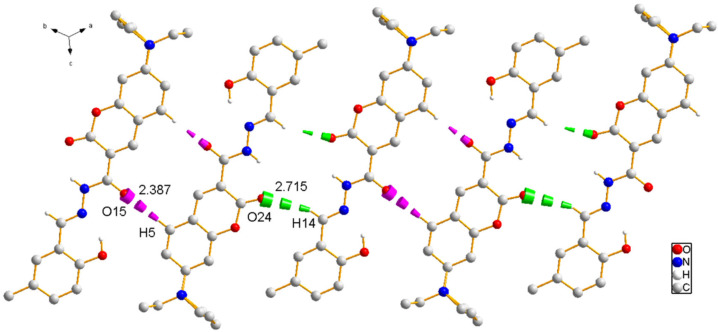
One-dimensional chain structure of compound **L** along the A-axis direction, formed through intermolecular C5-H5A…O15 (pink) and C14-H14…O24 (bright green) hydrogen bonding.

**Figure 4:**
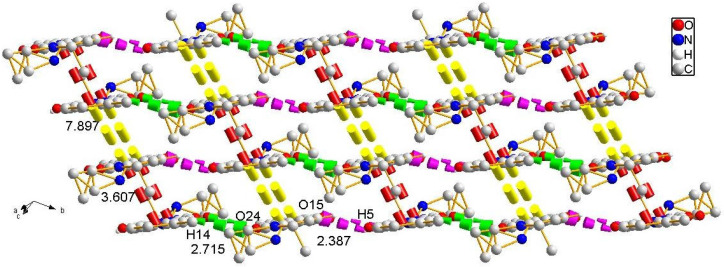
Two-dimensional planar structure of probe **L** along the C-axis direction, formed through π(P1)-π(P2) stacking (π-π shown in red and yellow).

Subsequently, the nucleophilic addition of intermediate II to 5-methylsalicylaldehyde yielded the acylhydrazone-based Schiff base probe **L** [5-methyl-2-hydroxybenzaldehyde-(7-diethylaminocoumarin-3-formyl)hydrazone] (Figure 1). Yellow needle-like crystals of **L** were obtained by slow evaporation of its saturated dichloromethane–cyclohexane (2:1, *v*/*v*) solution, and its structure was confirmed through single-crystal X-ray analysis. Compound **L** crystallized into a triclinic lattice with space group $P_{1}^{-}$. Through this analysis, the single-molecule diagram, one-dimensional diagram, two-dimensional diagram, and single-crystal data of **L** were acquired, as shown in Figure 2, Figure 3 and Figure 4 and Table 1, respectively. Each molecule of **L** is connected and grown through intermolecular hydrogen bonds (e.g., C5-H5A…O15 and C14-H14…O24) or π-π (P1-P2) stacking. These intermolecular forces also endow **L** with good optical properties [24].

### 3.2. Optical Properties of Fluorescent Probe ***L***

#### 3.2.1. Solvatochromism

**Figure 5:**
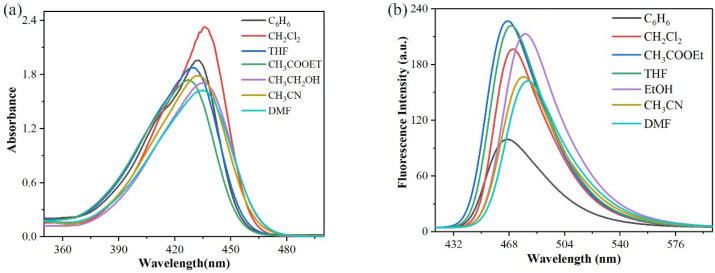
(**a**) Ultraviolet spectra of fluorescent probe **L** in different solvents; (**b**) fluorescence spectra of fluorescent probe **L** in different solvents.

As shown in Table 2 and Figure 5a, slight solvatochromism was observed with increasing solvent polarity, indicating that there was little difference in dipole moments between the ground and excited states of the chromophores, regardless of solvent polarity. In contrast, the fluorescence spectra of probe **L** in the seven tested solvents exhibited a significant red shift (Figure 5b), which indicated that solvent polarity exerted considerable influence on the fluorescence properties of probe **L**. This phenomenon can be attributed to the fact that the ground state of probe **L** is dominant in low-polarity solvents, whereas its excited state is dominant in high-polarity solvents. Thus, increased polarity resulted in a red shift in fluorescence emission [25]. The Stokes shift of probe **L** in different solvents exhibited a monotonic increase with the increase in solvent polarity, reaching the maximum value of 2155 cm^−1^ in DMF (Table 2). These results demonstrated that the molecule experienced the greatest energy loss in DMF through excited-state non-radiative transitions. This attribute endowed probe **L** with high detection sensitivity and low background interference [26].

#### 3.2.2. AIE Fluorescence Spectroscopy

**Figure 6:**
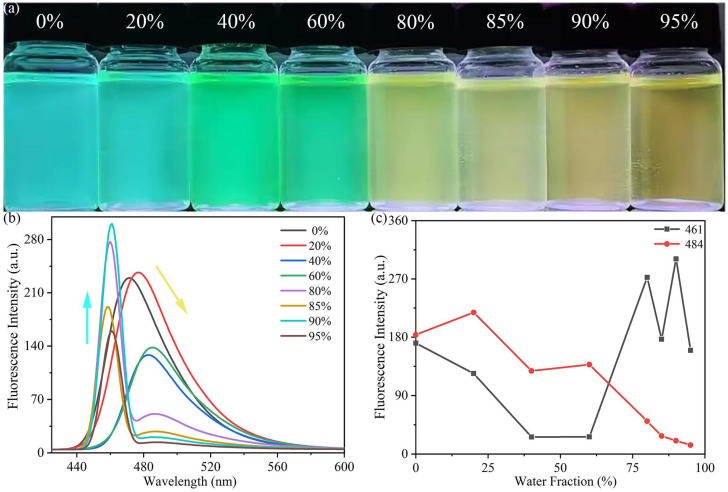
(**a**) Fluorescence changes in probe **L** at different water contents under 365 nm ultraviolet light irradiation; (**b**) fluorescence spectra of probe **L** in THF/water solvent mixtures of different ratios; (**c**) variation in fluorescence intensity of probe **L** with water content at wavelengths of 461 nm and 484 nm.

To further investigate the optical behavior of probe **L** during the aggregation process, good-solvent–poor-solvent fluorescence experiments were performed. THF was used as the good solvent and water was used as the poor solvent to obtain mixed solvent systems with water fractions (*f_w_*) ranging from 0% to 95%. As illustrated in Figure 6a, probe **L** displayed sky-blue fluorescence in pure THF solution. Its fluorescence color gradually turned green and finally turned yellow with the addition of water to THF, which was accompanied by a distinct red shift in the fluorescence spectra. Specifically, when the solvent system changed from pure THF to a THF/water mixture with a water fraction of 90%, the photoluminescence (PL) intensity at 461 nm increased from 170.7 arbitrary units (a.u.) to 300.8 a.u. In contrast, the fluorescence intensity at 484 nm decreased from 183.7 a.u. to 20.5 a.u. (Figure 6b,c), showing the characteristic of AIE. The underlying mechanism for these observations was likely as follows: In the good solvent, probe **L** molecules were in a dispersed state, with almost no interaction between them. Molecules could rotate freely around single bonds, which facilitates energy dissipation through non-radiative transitions, resulting in weak fluorescence intensity. However, when the poor solvent (water) was added, the aggregation of probe **L** molecules imposed spatial constraints on molecular motion and induced various intermolecular interaction forces, (e.g., C5-H5A…O15 and C14-H14…O24, as depicted in Figure 3 and Figure 4). This led to a significant enhancement in fluorescence intensity [27]. When the water content reached 95%, the fluorescence wavelength of probe **L** red-shifted to approximately 490 nm. This phenomenon is observed in many compounds with AIE properties and can be attributed to the increased aggregation of solute molecules in the solution into amorphous nanoparticles [28].

### 3.3. Recognition of Cu^2+^ by Fluorescent Probe ***L***

#### 3.3.1. Selectivity

**Figure 7:**
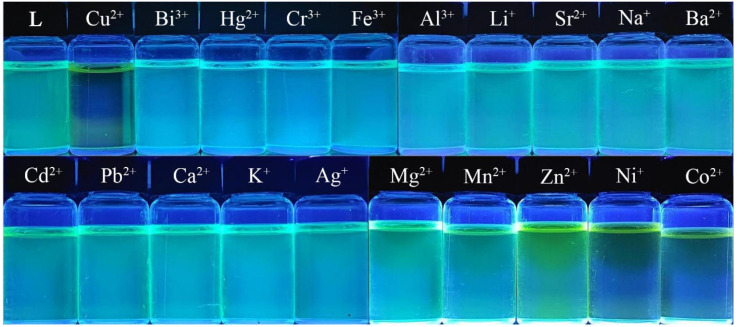
Photographs taken under 365 nm UV illumination after addition of 3 equiv. of various metal ions to probe **L**.

Selectivity is a crucial indicator for assessing the performance of fluorescent probes. The selective recognition ability of probe **L** was first tested visually by adding Cu^2+^ as well as various other metal ions to a solution of probe **L** under UV illumination. As seen from Figure 7, the solution color of probe **L** changed from cyan to colorless under 365 nm UV light upon addition of Cu^2+^, while no significant color change was observed with other ions (except for a slight change with Ni^2+^ and Co^2+^). This phenomenon can be ascribed to the enhanced metal–ligand charge-transfer (MLCT) effect between probe **L** and Cu^2+^ upon their complexation, which in turn induced the chelation-enhanced fluorescence quenching (CHEQ) effect [29].

**Figure 8:**
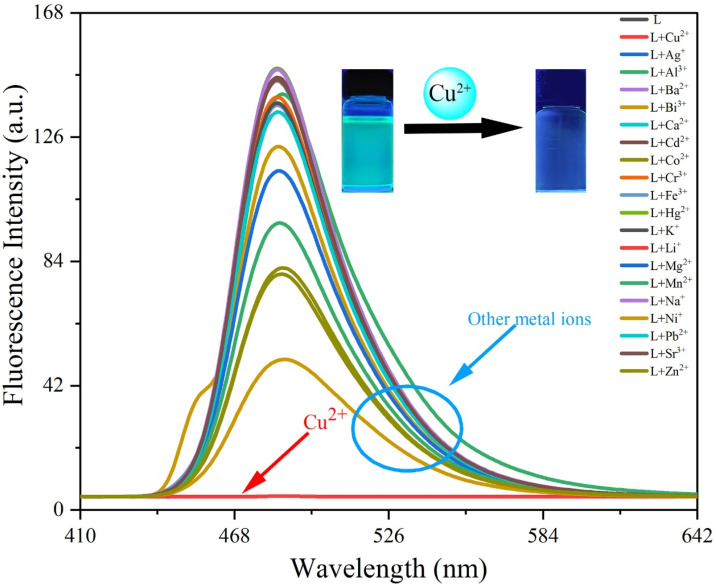
Fluorescence spectra of probe **L** after addition of various metal ions.

**Figure 9:**
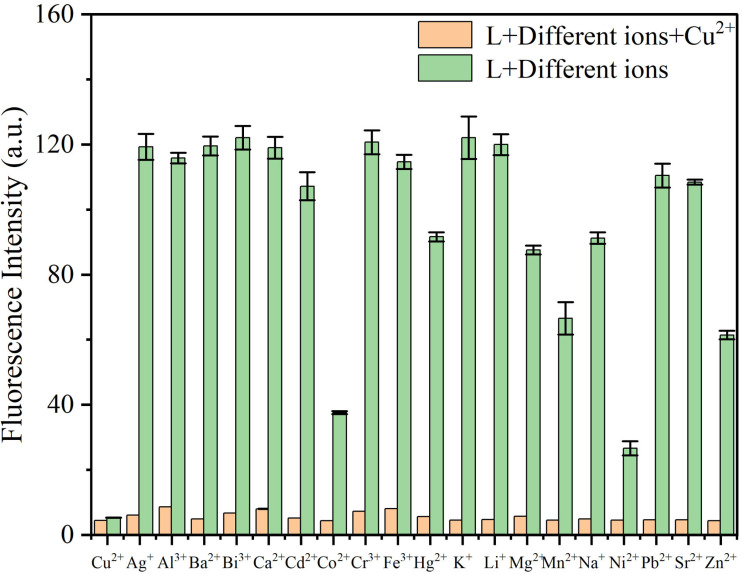
Interference of other co-existing metal ions on the recognition of Cu^2+^ by probe **L**.

To further investigate the recognition behavior of probe **L** toward different metal ions, the fluorescence emission spectra of **L** were recorded in DMSO. The results showed that only Cu^2+^ caused a significant reduction in the fluorescence emission intensity at 484 nm (from 147.5 a.u. to 4.72 a.u.), corresponding to a 31.3-fold reduction. In contrast, other ions caused negligible changes, except for Ni^2+^ and Co^2+^ (Figure 8). The reduction in fluorescence with the addition of Ni^2+^ and Co^2+^ ions was only 2.92-fold and 1.84-fold, respectively, indicating that probe **L** could discriminate between Cu^2+^ and these two ions. The selectivity was also visually evident, which was confirmed by the following anti-interference experiment (Figure 9).

Another critical parameter for fluorescent probes is response speed, which is key to achieving sensitive responses in practical applications. Upon adding three equivalents of Cu^2+^ to probe **L**, the fluorescence intensity reduced rapidly and reached the minimum value within 15 s (Figure S6). The above results collectively demonstrate the excellent selectivity of probe **L** for Cu^2+^ with a rapid response toward Cu^2+^.

#### 3.3.2. Anti-Interference Testing

To further evaluate the selectivity of probe **L** as a Cu^2+^-specific fluorescence chemosensor and verify its anti-interference capability, a co-existing ion competition experiment was conducted [30]. Several common interferents, including biologically and environmentally relevant ions such as Zn^2+^, Fe^3+^, Hg^2+^, and Pb^2+^, were selected for this experiment. As shown in Figure 9, the fluorescence reduction due to Cu^2+^ was barely affected by other metal ions. Even in the presence of 19 other ions (three equiv.), copper ions efficiently reduced the fluorescence of **L**, indicating good selectivity for Cu^2+^.

#### 3.3.3. Ultraviolet Titration Experiments

**Figure 10:**
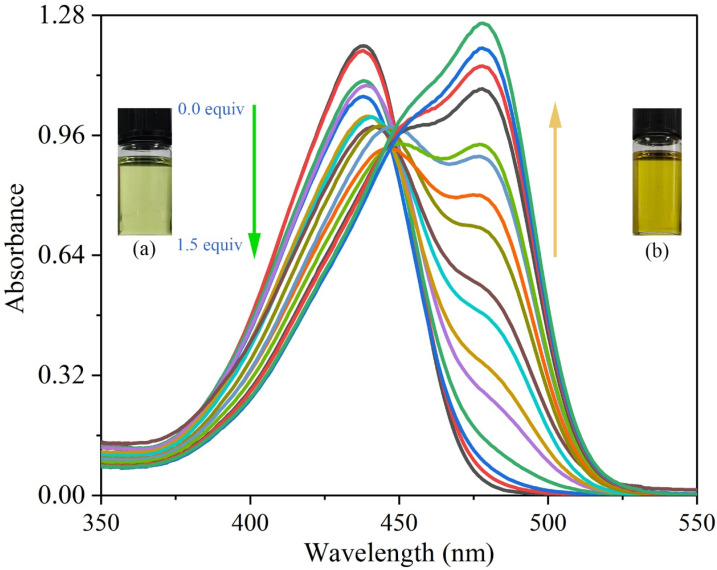
Ultraviolet spectrum changes after gradual addition of Cu^2+^. The inset depicts (a) light green in the absence of Cu^2+^ and (b) yellow in the presence of 1.5 equiv. of Cu^2+^ under daylight.

To investigate the photophysical properties of probe **L** in the presence and absence of Cu^2+^ ions, UV–Vis titration experiments were performed (Figure 10) [31]. In the absence of metal ions, probe **L** displayed a broad absorption band at 438 nm in DMSO. Upon the gradual addition of Cu^2+^ ions to the solution of **L**, the absorption band at 438 nm gradually diminished, and a new band emerged at 478 nm; concurrently, the color of the solution changed from light green to yellow, as the inset depicts. The emergence of the new peak at 478 nm was attributed to the coordination of Cu^2+^ with probe **L**. The isolated electron pairs on the imino group also bind to the metal ions, resulting in red shifts in both excitation and emission bands. Similar results have been reported for several organic moieties in the presence of Cu^2+^ ions [32,33]. Thus, it was speculated that intramolecular charge transfer played a crucial role in the UV–Vis spectral changes in probe **L** upon the addition of Cu^2+^ ions. Additionally, when the concentration of Cu^2+^ reached 1.33× 10^−5^ mol/L, the system attained equilibrium, with an isosbestic point appearing at 448 nm. Therefore, probe **L** can be utilized as a selective on–off-type fluorescence sensor for Cu^2+^ [34,35].

#### 3.3.4. Fluorescence Titration Experiments and Detection Limit

**Figure 11:**
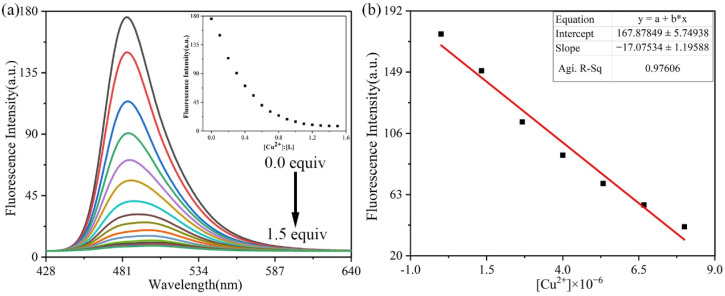
(**a**) Fluorescence spectrum changes after gradual addition of Cu^2+^; (**b**) slope of the titration standard curve.

To investigate the recognition sensitivity of probe **L** towards Cu^2+^, a quantitative analysis was conducted using fluorescence titration in DMSO. As illustrated in Figure 11a, the fluorescence intensity systematically decreased and red-shifted with the increase in Cu^2+^ concentration from 0 to 1.67 × 10^−5^ mol/L, reaching complete fluorescence quenching at 1.33 × 10^−5^ mol/L. The results suggested that the binding ratio of probe **L** to Cu^2+^ was 1:1. A good linear correlation (R^2^ = 0.97606) was established between the fluorescence intensity at 484 nm and Cu^2+^ concentration in the range of 0~8 × 10^−6^ mol/L (Figure 11b). The linear regression equation was y = −17x + 167.9. Based on this favorable linear relationship, further studies were conducted to determine the minimum detection limit of probe **L** for Cu^2+^ (Figure S7). The limit of detection (LOD) of probe **L** was calculated to be 7.8 × 10^−7^ mol/L, which is much lower than the maximum LOD specified by the World Health Organization (30 μM) [36]. Moreover, as shown in Table S3, the copper-sensing performance of probe L was compared with other Cu^2+^ detection probes [37,38]. It can be seen that probe **L** exhibited high sensitivity and a fast response, indicating comparable and even superior performance to other probes.

#### 3.3.5. Complexation Ratio and Binding Constant

**Figure 12:**
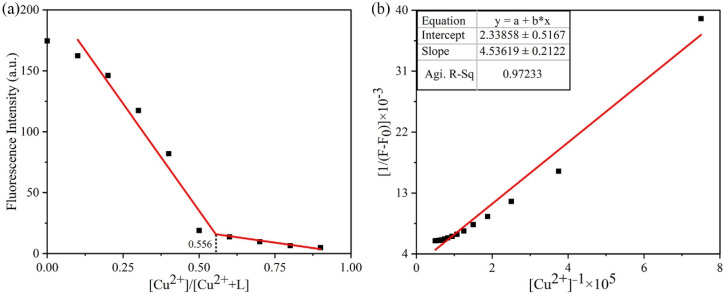
(**a**) Job’s plot of probe **L** and Cu^2+^**;** (**b**) Benesi–Hildebrand equation plot of probe **L** and Cu^2+^.

To determine the binding stoichiometry, Job’s continuous variation experiment was performed by monitoring fluorescence intensity at 484 nm as a function of the mole fraction [Cu^2+^]/[L+ Cu^2+^] [39]. The molar ratio of Cu^2+^ was varied from 0.1 to 0.9 while the total solution volume was kept constant at 200 μL. At a ratio of 0.556, Job’s plot (Figure 12a) showed negligible change in the fluorescence emission intensity of probe **L**, essentially reaching the minimum value. This result indicated 1:1 stoichiometric binding of Cu^2+^to **L**, which was further confirmed by fluorescence titration experiments and crystal structure analysis of the **L**-Cu^2+^ complex.

By monitoring the variations in the fluorescence intensity of probe **L** with different concentrations of Cu^2+^ at room temperature, the apparent binding constant (K_b_) was determined using the Benesi–Hildebrand plot (Figure 12b). The linear equation was y = 4.54x + 2.34, and the linear correlation coefficient was 0.97233. The binding constant of probe **L** with Cu^2+^ was calculated as K_b_ = 5.2 × 10^6^ L/mol [40,41], which verified a strong binding interaction.

#### 3.3.6. Binding Behavior of Cu^2+^ with Probe **L**

**Figure 13:**
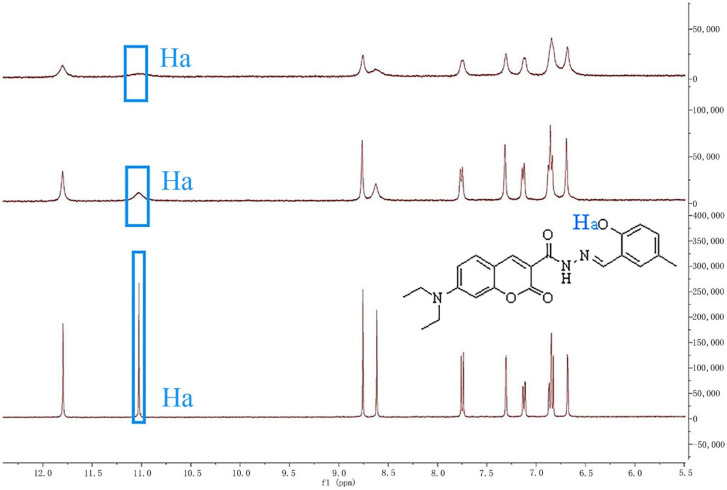
^1^H-NMR data of probe **L** in DMSO-d_6_ solution in the absence and presence of Cu^2+^.

To investigate the recognition mechanism of probe **L** for Cu^2+^, an ^1^H NMR titration experiment was performed to explore the interaction between probe **L** and Cu^2+^ [42,43,44]. Since Cu^2+^ is a paramagnetic ion, it is not suitable for quantitative NMR experiments. Thus, only qualitative NMR was employed herein. According to Figure 13, upon addition of Cu^2+^ to the DMSO-d_6_ solution of **L**, the ^1^H NMR peak corresponding to the -OH group (at a chemical shift of *δ* = 11.01 ppm) gradually decreased and eventually nearly disappeared. In contrast, the chemical shifts in other major ^1^H NMR peaks remained unchanged. This observation indicated that Cu^2+^ coordinated with the phenolic hydroxyl group of probe **L**, resulting in the disappearance of the hydrogen atom signal. The above phenomenon can be attributed to the CHEQ effect [45], which arises from MLCT between Cu^2+^ and the phenolic hydroxyl group of probe **L**.

**Figure 14:**
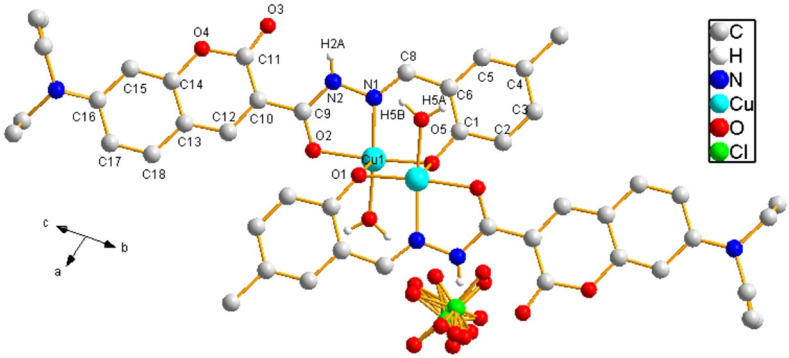
Single molecular structure of coordination compound **CuLClO_4_**.

Probe **L** possesses multiple coordination sites. To elucidate its coordination mode, crystals of its complexes with Cu^2+^ were obtained by layered diffusion (Scheme S1). The Cu^2+^ complex is a centrosymmetric dinuclear complex crystallized in the triclinic system and $P_{1}^{-}$ space group. C-O bond distances of 1.263(3) and 1.332(3)Å for C(9)-O(2) and C(1)-O(1), respectively, together with 1.342(3) for C(9)-N(2) and 1.290(3) Å for C(8)-N(1), suggest that the proton on O(1) is lost and **L** acts as a monoanionic ligand during coordination (Table S2) [46]. In the asymmetric unit of the Cu^2+^ complex, the central Cu^2+^ resides in a five-coordinate environment, chelating with one deprotonated **L** through two O atoms and one N atom, another deprotonated **L** through one O atom, and one H_2_O molecule through one O atom, forming a square pyramid coordination geometry. Interestingly, residual electron density at the phenolic oxygen attracts one additional electron-deficient Cu^2+^ center, forming a centrosymmetric dinuclear complex with an oxygen-bridge structure, which is positively charged as a whole. Finally, it interacts with ClO_4_^−^ to form an electrically neutral complex in solution. As seen from Figure 14, N1 and O2 on the formylhydrazone moiety of probe **L**, O5 in water, and O1 atom on the phenolic hydroxyl group of two probe **L** molecules form a rare five-coordinate chelate with Cu^2+^. This strongly confirmed that Cu^2+^ coordinated with the classical recognition structure (Schiff base structure) of **L** in a 1:1 stoichiometry, leading to a CHEQ effect resulting from MLCT. The above conclusion is consistent with the results of UV–Vis, fluorescence titration and Job’s plot of Cu^2+^.

## 4. Conclusions

In summary, a fluorescent probe with AIE properties was developed to selectively recognize Cu^2+^. The addition of Cu^2+^ triggered an immediate and pronounced reduction in fluorescence intensity. Comprehensive investigations, including fluorescence titration, single-crystal X-ray diffraction, and Job’s plot analysis, confirmed a 1:1 (Cu^2+^: L) binding model. The coordination process induced chelation-induced fluorescence quenching, attributed to metal-to-ligand charge transfer. Quantitative analysis based on fluorescence titration revealed a binding constant (K_b_) of 5.2 × 10^6^ L/mol and a detection limit of 7.8 × 10^−7^ mol/L, highlighting the probe’s excellent sensitivity. These findings suggest that probe **L** is a promising candidate for environmental monitoring applications, providing an effective technical means for Cu^2+^ detection and analysis, with significant implications for environmental protection and public health safety. Further studies are underway to expand the scope and applicability of this probe for detection of metal ions under various biologically and environmentally relevant conditions.

## Figures and Tables

**Table 1:** Crystallographic data of compound **L** and **CuLClO_4_**.

Compounds	L *	CuLClO_4_ **
Molecular formula	C_22_H_23_N_3_O_4_	C_44_H_52_Cl_2_Cu_2_N_6_O_20_
Molecular weight	393.43	1182.89
Crystal system	triclinic	triclinic
Space group	$P_{1}^{-}$	$P_{1}^{-}$
a [Å]	8.6975(3)	10.3929(4)
*b* [Å]	8.7140(3)	11.0883(5)
*c* [Å]	14.8110(4)	12.2930(3)
*α*[°]	102.576(3)	106.301(3)
*β*[°]	93.554(2)	92.083(3)
*γ*[°]	112.329(3)	113.075(4)
*V* [Å^3^]	1000.34(6)	1233.60(9)
*Z*	2	1
*D*calcd [g cm^−3^]	1.306	1.592
*μ* [mm^−1^]	0.091	0.760
no. params refined	3704	7271
*R*_1_, *wR*_2_*(I ≥* 2*σ (I))*	0.049, 0.1380	0.0439, 0.1128
*R*_1_, *wR*_2_*(all data)*	0.0901, 0.1606	0.0564, 0.1206
GOF	0.995	1.068

* CCDC No: 2413588; ** CCDC No: 2413597.

**Table 2:** UV–Vis and fluorescence assay results of probe **L** in different solvents.

Solvent	λmax ^a^ (ε_max_ ^b^)	λ_max_ ^c^ (I_max_ ^d^)	Δν ^e^
C_6_H_6_	433(1.97)	467(99.54)	1470
CH_2_Cl_2_	436(2.33)	470(196.7)	1659
CH_3_COOEt	429(1.72)	467(227.0)	1897
THF	427(1.86)	469(221.9)	2097
EtOH	436(1.71)	478(213.0)	2015
CH_3_CN	433(1.79)	477(166.9)	2130
DMF	435(1.62)	480(164.5)	2155

Note: ^a^ Peak position of the largest absorption band in nm (1.0 × 10^−5^ mol/L); ^b^ maximum molar absorbance in 10^4^ L/(mol·cm); ^c^ peak position of single-photon emission fluorescence (SPEF), excited at the absorption maximum; ^d^ fluorescence intensity at the two different emission peaks; ^e^ Stokes shift in cm^−1^.

## Data Availability

The data presented in this study are available upon request from the corresponding author.

## Supplementary Materials

The following supporting information can be downloaded at: https://www.mdpi.com/article/10.3390/s26072087/s1, Figure S1: Molecular structure of intermediate I; Figure S2: Molecular structure of intermediate II; Table S1: Crystal data for intermediate I and intermediate II; Figure S3: Infrared spectra of compound **L**; Figure S4: ^1^H-NMR (400 MHz, CDCl_3_) spectrum of compound **L**; Figure S5: ^13^C-NMR (100 MHz, CDCl_3_) spectrum of compound **L**; Figure S6: Fluorescence intensity variation with time after adding three equiv. of Cu^2+^ to probe **L**; Figure S7: Fluorescence spectra of **L** without Cu^2+^ measured twenty times; Scheme S1: Synthesis of Cu(ClO_4_)_2_ **L**; Table S2: Selected bond lengths (Å), bond angles (o) and dihedral angles of **L** and **CuLClO_4_**; Table S3: Comparison of representative Cu^2+^ fluorescent sensors with the present work. References [21,47,48,49,50,51,52,53] are cited in the Supplementary Materials.

## References

[1] Briffa J., Sinagra E., Blundell R. (2020). Heavy metal pollution in the environment and their toxicological effects on humans. Heliyon..

[2] Zhu Z., Song M., Ren J., Liang L., Mao G., Chen M. (2024). Copper homeostasis and cuproptosis in central nervous system diseases. Cell Death Dis..

[3] Jukkrit N., Rathawat D., Chanchai S., Worawat W., Suttipong W., Paitoon R., Kantapat C. (2021). The synergy of CHEF and ICT toward fluorescence ‘turn-on’ probes based on push-pull benzothiazoles for selective detection of Cu^2+^ in acetonitrile/water mixture. J. Photochem. Photobiol. A..

[4] Zhang Y., Lu P., Peng P., Wei J., Shi W., Lu L., Zhou Q., Pu Y., Yin L. (2025). Acute Cu exposure induces neurotoxicity via DAF-16/FoxO and SKN-1/Nrf2 pathway. J. Environ. Sci..

[5] Oe S., Miyagawa K., Honma Y., Harada M. (2016). Copper induces hepatocyte injury due to the endoplasmic reticulum stress in cultured cells and patients with Wilson disease. Exp. Cell Res..

[6] Yao Y., Long B., Zhu M., Zhang S., Liu H., Tian L. (2025). Effects of BDE3 and the Co-Existence Copper on Photosynthesis and Antioxidative Enzymes in *Salvinia natans* (L.). Water..

[7] Karakebap K., Serbest H., Turak F., Bakırdere S. (2025). Trace copper determination in mate tea and tap water using FAAS and spray-assisted liquid phase microextraction. J. Food Compos. Anal..

[8] Gosavi P.M., Gawali S.S., Lande D.N., Gejji S.P., Butcher R.J. (2025). Ethylaminoanthraquinone-based optical sensor for Cu^2+^. J. Mol. Struct..

[9] Ören S., Özbek O., Isildak Ö. (2024). The use of thiazole derivative molecules as sensor materials: Potentiometric determination of Cu(II) ions. Vietnam J. Chem..

[10] Kizil N., Uzcan F., Beydagi B.B., Sahin M., Tokum B.A., Yola M.L., Soylak M. (2025). Determination of copper (II) in seafood and water samples by ICP-OES using magnetic titanium aluminum carbide nanocomposite for solid phase microextraction. J. Food Compos. Anal..

[11] Hou J., Xu X., Wang Z., Yang X., Rao X., Zhao P., Jiang Q. (2025). Dicyanoisophorone-based fluorescence probe for detection of Cu^2+^ and its applications in living cells and mice. Spectrochim. Acta A..

[12] Musikavanhu B., Huang X., Ma Q., Xue Z., Feng L., Zhao L. (2024). Rhodamine-benzothiazole-thiophene: A triangular molecular tool for simultaneous detection of Hg^2+^ and Cu^2+^. Microchem. J..

[13] Sushil K., Siddhant S., Arun K., Pramod K. (2021). Recognition, mechanistic investigation and applications for the detection of biorelevant Cu^2+^/Fe^2+^/Fe^3+^ ions by ruthenium(II)-polypyridyl based fluorescent sensors. Dalton Trans..

[14] Jiang H., Li Z., Kang Y., Ding L., Qiao S., Jia S., Luo W., Liu W. (2017). A two-photon fluorescent probe for Cu^2+^ based on dansyl moiety and its application in bioimaging. Sens. Actuators B.

[15] Yu C., Huang J., Yang M., Zhang J. (2024). Construction of Chitosan-Modified Naphthalimide Fluorescence Probe for Selective Detection of Cu^2+^. Sensors..

[16] Mayurachayakul P., Chantarasriwong O., Kamkaew A., Sukwattanasinitt M., Niamnont N. (2024). A novel triphenylamine-furan hydrazone-based sensing of Cu^2+^ ions and imaging in cancer cells. J. Mol. Struct..

[17] Zavalishin M.N., Gamov G.A., Kiselev A.N., Nikitin G.A. (2024). A fluorescein conjugate as colorimetric and red-emissive fluorescence chemosensor for selective recognition Cu^2+^ ions. Opt. Mater..

[18] Pichayanan S., Anirut S., Wutthinan T., Teerapong J., Varomyalin T., Nararak L., Dhassida S. (2022). Highly sensitive and selective coumarin-based fluorescent chemosensor for Cu^2+^ detection. J. Photochem. Photobiol. A..

[19] Mani K.S., Rajamanikandan R., Murugesapandian B., Shankar R., Sivaraman G., Ilanchelian M., Rajendran S.P. (2019). Coumarin based hydrazone as an ICT-based fluorescence chemosensor for the detection of Cu^2+^ ions and the application in HeLa cells. Spectrochim. Acta Part A..

[20] Niranjan R., Prasad G.D., Arockiaraj M., Rajeshkumar V., Sundramoorthy A.K., Mahadevegowda S.H. (2025). A Coumarin-Julolidine conjugated novel Schiff base: Synthesis, DFT insights, and evaluation of selective and sensitive dual-responsive fluorescence sensing capabilities with Cu^+^ and Cu^2+^ ions. Chem. Phys..

[21] Ma Q., Yang X., Zhao Y. (2025). Development of a Coumarin-Based Schiff Base Fluorescent Probe and its Application in Detection of Cu^2+^. J. Fluoresc..

[22] Liu J., Chen Q., Li C., Yu Q., Li Z., Wang L., Wu X., Zhang Z. (2024). Synthesis of aggregation-induced emission fluorescent probe and the application in recognition of Al^3+^. Chin. J. Anal. Lab..

[23] Liu J., Li C., Chen Q., Li Q., Li C., Li S., Zhang Z., Xu L. (2022). A Novel AIE Fluorescent Probe for Cu^2+^ Recognition Based on Salicylaldehyde-azine System. ChemistrySelect.

[24] Yang X., Zhang W., Yang Z., Xu H., Wu J., He L. (2017). Highly sensitive and selective fluorescent sensor for copper(ii) based on salicylaldehyde Schiff-base derivatives with aggregation induced emission and mechanoluminescence. New J. Chem..

[25] Katritzky A.R., Fara D.C., Yang H., Tämm K., Tamm T., Karelson M. (2004). Quantitative measures of solvent polarity. Chem. Rev..

[26] Wang Y., Wang J., Liu R., Chen J., Shu Y., Wang J., Qiu H. (2024). Highly selective near-infrared fluorescent turn-on probe with a large Stokes shift for the detection of hydrogen sulfide and imaging in live cells. Microchem. J..

[27] Hong D., Feng Y., Wan J., Yang W., Gao L., Zhao J., Ya H., Liu G., Li Z. (2025). Reinforced donor-acceptor type dithienylethene with aggregation-induced emission for visible light-triggered photoswitching behavior in aqueous media. J. Mol. Struct..

[28] Rauf A., Shah A., Munawar K.S., Ali S., Tahir M.N., Javed M., Khan A.M. (2020). Synthesis, physicochemical elucidation, biological screening and molecular docking studies of a Schiff base and its metal(II) complexes. Arab. J. Chem..

[29] Harathi J., Thenmozhi K. (2020). AIE-active Schiff base compounds as fluorescent probes for the highly sensitive and selective detection of Fe^3+^ ions. Mater. Chem. Front..

[30] Wang X., Shi W., Feng L., Ma J., Li Y., Kong X., Chen Y., Hui Y., Xie Z. (2017). A highly selective and sensitive Schiff-base based turn-on optical sensor for Cu^2+^ in aqueous medium and acetonitrile. Inorg. Chem. Commun..

[31] Liu L., Shen B., Yang H., Zhu H., Feng X., Zhang R., Hong S., Gao H. (2024). Acylhydrazone-functionalized triphenylamine V-shaped liquid crystals: Synthesis, columnar self-assembly, gel property and application for the detection of Cu^2+^. J. Mol. Liq..

[32] Hong M.C., Sangkyun N., Eun S.J., Kyun K.J., Geun J.T., Cheal K. (2017). A new Schiff-based chemosensor for chromogenic sensing of Cu^2+^, Co^2+^ and S^2−^ in aqueous solution: Experimental and theoretical studies. New J. Chem..

[33] Zhang S., Niu Q., Lan L., Li T. (2017). Novel oligothiophene-phenylamine based Schiff base as a fluorescent chemosensor for the dual-channel detection of Hg^2+^ and Cu^2+^ with high sensitivity and selectivity. Sens. Actuators B Chem..

[34] Hu H., Li J., Shi W., Jing T., Zhang C., Gao C., Sun C., Du Y., Hu B. (2023). A series of novel 1H-indole-7-carbohydrazide derivatives with photoswitching and AIE properties: “On-off” fluorescence sensors for Cu^2+^. J. Mol. Struct..

[35] Cao X., You J., Liu Q., Liu B., Yu Y., Wu W. (2024). A dual-functional fluorescent sensor based on dihydropyrazole derivative for successive detection of Cu^2+^ and glyphosate and its applications. Mater. Today Commun..

[36] Olaciregui J.P., Sesto E.V., Taton D., Pomposo J.A. (2024). Lanthanide-based Single-Chain Nanoparticles as “Visual” Pass/Fail Sensors of Maximum Permissible Concentration of Cu^2+^ Ions in Drinking Water. Macromol. Rapid Commun..

[37] Li C.H., Zhao S.S., Liu X.X., Zheng Y.H., Liu H.Y., Li H.Y. (2025). Phosphorescent iridium(III) complexes featuring Ir-S-C-S structures as chemosensors for selective recognition of Cu^2+^ ions. J. Lumin..

[38] Aarjane M., Slassi S., Amine A. (2020). Novel highly selective and sensitive fluorescent sensor for copper detection based on N -acylhydrazone acridone derivative. J. Mol. Struct..

[39] Guo X., Guo C., Xing Y., Liu Y., Wei K., Kang M., Yang X., Pei M., Zhang G. (2022). A novel Schiff base sensor through “off-on-off” fluorescence behavior for sequentially monitoring Al^3+^ and Cu^2+^. J. Photochem. Photobiol. A..

[40] Wei C.P., Piew H.M., Mat S.H., Shin S.K., Wai T.K. (2021). Specific detection of Cu^2+^ by a pH-independent colorimetric rhodamine based chemosensor. Opt. Mater..

[41] Vyas S., Barot Y.B., Mishra R. (2024). Novel Anthracene and Carbazole Based Aggregation Induced Enhanced Emission Active Schiff Base as a Selective Sensor for Cu^2+^ ions. J. Fluoresc..

[42] Lu L., Hanshu Z., Yun G., He Z., Hanyan Y., Ruilin Z., Yu Y., Hongfei G. (2023). Pyrene-acylhydrazone-based Turn-on Fluorescent Probe for Highly Sensitive Detection Cu^2+^ and application in Bioimaging. J. Fluoresc..

[43] Kumar A., Mohan B., Solovev A.A., Saini M., Sharma H.K. (2022). Development of 2-hydroxy-naphthaldehyde functionalized Schiff base chemosensor for spectroscopic and colorimetric detection of Cu^2+^ and Pd^2+^ ions. Microchem. J..

[44] Chen M., Cao F., Huang S., Li Y., Zhong M., Zhu M. (2022). The schiff base probe with j-aggregation induced emission for selective detection of Cu^2+^. J. Fluoresc..

[45] Hamzi I. (2024). Colorimetric and Fluorometric N-Acylhydrazone-based Chemosensors for Detection of Single to Multiple Metal Ions: Design Strategies and Analytical Applications. J. Fluoresc..

[46] Fu S., Zhang G., Wang N., Yang J., Li J., Tian S., Wu S., Yin F., Chen C., Yang Q. (2025). A novel long-alkyl-chained Schiff bases supramolecular Self assembled material for ultrasensitive detection of Fe^3+^ and Cu^2+^. J. Mol. Struct..

[47] Pang S., Yu Y., Yan X., Wu M., Liu Q., Zu P., Wu C. (2024). Synthesis of Coumarinylhydrazone Fluorescent Probe and its Relay Recognition of Cu^2+^ and HPO_4_^2−^. J. Fluoresc..

[48] Tang Y., Li Y., Han J., Mao Y., Ni L., Wang Y. (2018). A coumarin based fluorescent probe for rapidly distinguishing of hypochlorite and copper (II) ion in organisms. Spectrochim. Acta A.

[49] Wang Y., Hao X., Liang L., Gao L., Ren X., Wu Y., Zhao H. (2020). A coumarin-containing Schiff base fluorescent probe with AIE effect for the copper(II) ion. RSC Adv..

[50] Tahir S., Abdurrahman K., Nihan K.E.Ş., Duygu A., Furkan Ö., Kenan K., Nur A.F., Orhan G.A., İbrahim Y. (2020). Fluorescent sensing platform for low-cost detection of Cu^2+^ by coumarin derivative: DFT calculation and practical application in herbal and black tea samples. Turk. J. Chem..

[51] Abbas W.R., Kadhim M.A. (2025). Design and synthesis of new coumarin-based fluorescent chemosensors for the dual detection of Hg^2+^ and Cu^2+^ in aqueous and biological samples. RSC Adv..

[52] García-Beltrán O., Cassels B., Pérez C., Mena N., Núñez M., Martínez N., Pavez P., Aliaga M. (2014). Coumarin-Based Fluorescent Probes for Dual Recognition of Copper(II) and Iron(III) Ions and Their Application in Bio-Imaging. Sensors.

[53] Li K., Huang Y., Sun Y., Zhang Y., Zhang Y., Ren B., Cao D. (2025). A hydroxyl coumarin-chalcone-based fluorescent probe for sensing copper ions in plant and living cells. J. Photoch. Photobio.B..

