# Supplementary material for: Highly Selective and Sensitive Fluorescent Probe for Copper (II) Ions Based on Coumarin Derivative with Aggregation-Induced Emission
Source: Sensors (Basel). 2026 Mar 27;26(7):2087. doi: 10.3390/s26072087 (PMC13075019; doi:10.3390/s26072087)
Supplement: Supplementary file 1 [file sensors-26-02087-s001.zip › sensors-4187234-supplementary.pdf]

Electronic Supplementary Information

A highly selective and sensitive fluorescent probe for copper (II) ions based on coumarin derivative with aggregation-induced emission

JieLiu[1,*], PengChen[1],GuoyuGuo[1], XinboGao[1], YaozuXie[1], Zikang Li[1], Zhen Zhang[1] and Shuisheng Chen[1]

(Anhui Provincial Key Laboratory of Green Carbon Chemistry, School of Chemistry and Material Engineering, Fuyang Normal University, Fuyang 236037, China)

Table of Contents

Figure S1 Molecular structure of intermediate I 2

Figure S2 Molecular structure of intermediate II 2

Table S1 Crystal data for intermediate I and intermediate II 3

Figure S3 Infrared spectra of compound **L** 3

Figure S4 ^{1}H-NMR (400 MHz, $CDCl_3$) spectrum of compound **L** 4

Figure S5 ^{13}C-NMR (100 MHz, $CDCl_3$) spectrum of compound **L** 4

Figure S6 Fluorescence intensity variation with time after adding 3 equiv of Cu^{2+} to probe **L**. 5

Figure S7 Fluorescence spectra of **L** without Cu^{2+} measured twenty times............................. 5

Scheme S1 Synthesis of $Cu(ClO_4)_2$ **L** 6

Table S2 Selected Bond Lengths (Å), Bond Angles (°) and Dihedral Angles of **L** and **$CuLClO_4$**.. 6

Table S3 Comparison of representative Cu^{2+} fluorescent sensors with the present work 7

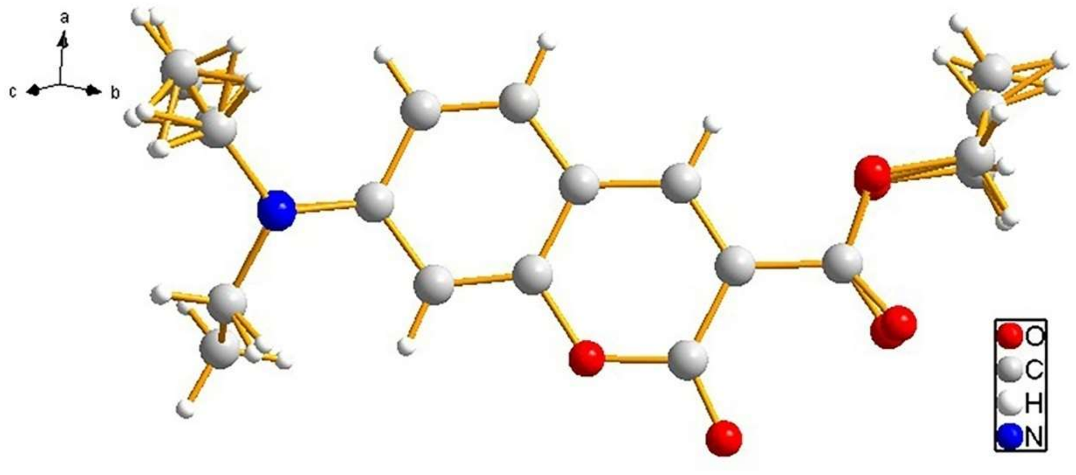

Figure S1 Molecular structure of intermediate I

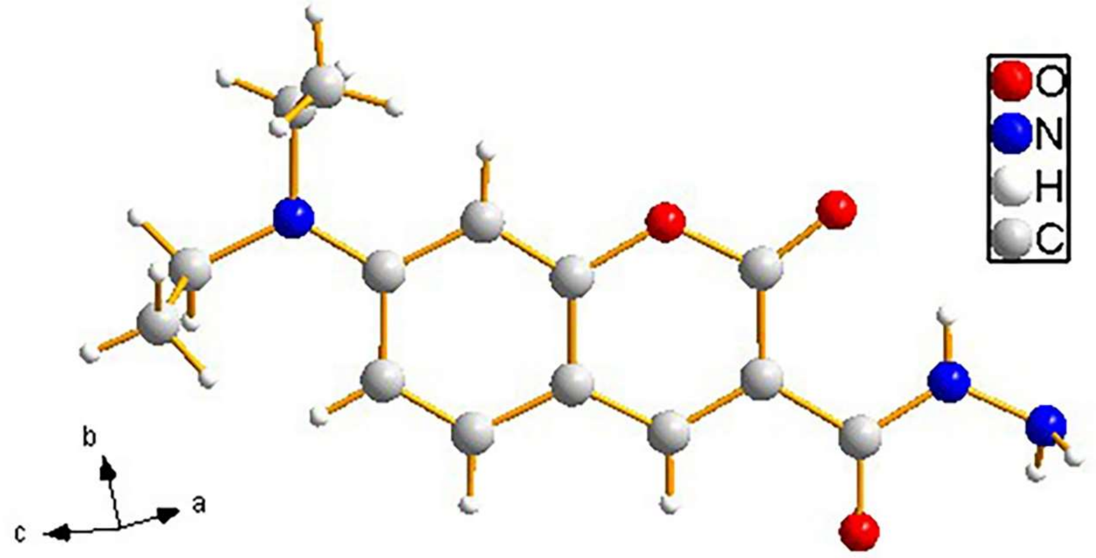

Figure S2 Molecular structure of intermediate II

Table S1 Crystal data for intermediate I and intermediate II

Compounds	intermediate I*	intermediate II**
Empirical Formula	$C_{16}H_{19}NO_4$	$C_{14}H_{17}N_3O_3$
Formula weight	289.32	275.30
Crystal system	monoclinic	triclinic
Space group	$P2_1/c$	$P\bar{1}$
a [Å]	6.8120(3)	9.3330(3)
b [Å]	15.9743(7)	12.7830(5)
c [Å]	13.6960(6)	12.9892(5)
α[°]	90	95.187(3)
β[°]	95.282(4)	110.118(3
γ[°]	90	106.076(3)
V [Å^3]	1484.03(11)	1368.29(10)
Z	4	4
D_{calcd}[g ·cm^{-3}]	1.295	1.336
μ [mm^{-1}]	0.093	0.096
no. params refined	3667	6746
R_1, wR_2 $(I \geq 2\sigma (I))$	0.0453, 0.1157	0.0528,0.1469
R_1, wR_2 (all data)	0.0739, 0.1299	0.0802,0.1663
GOF	1.066	1.068

* CCDC No :2126882 **CCDC No:2413945

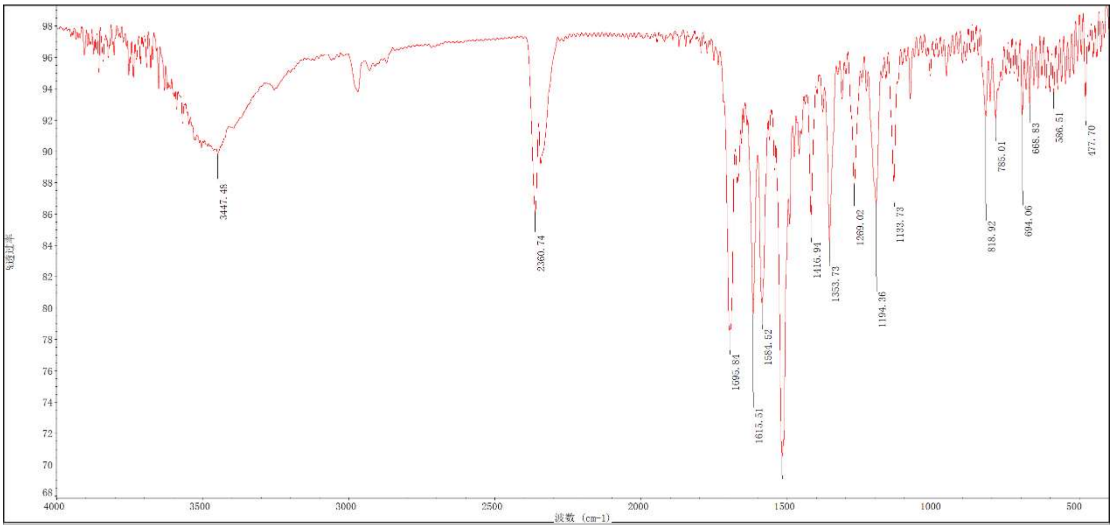

Figure S3 Infrared spectra of compound **L**

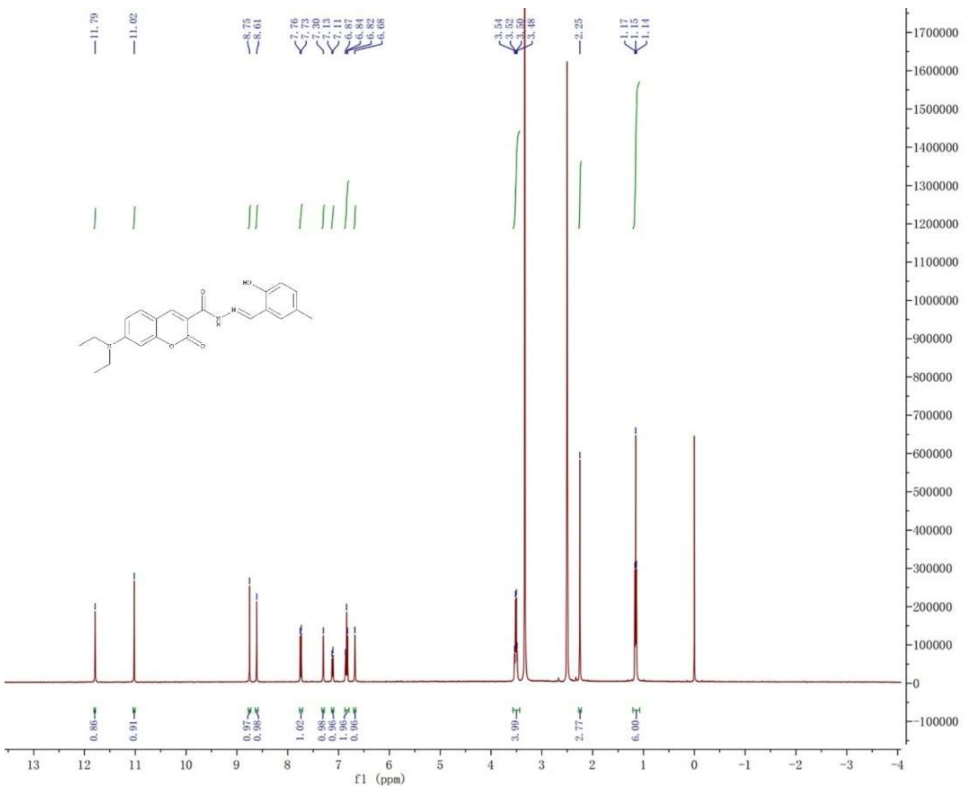

Figure S4 ^{1}H-NMR (400 MHz, $CDCl_3$) spectrum of compound **L**

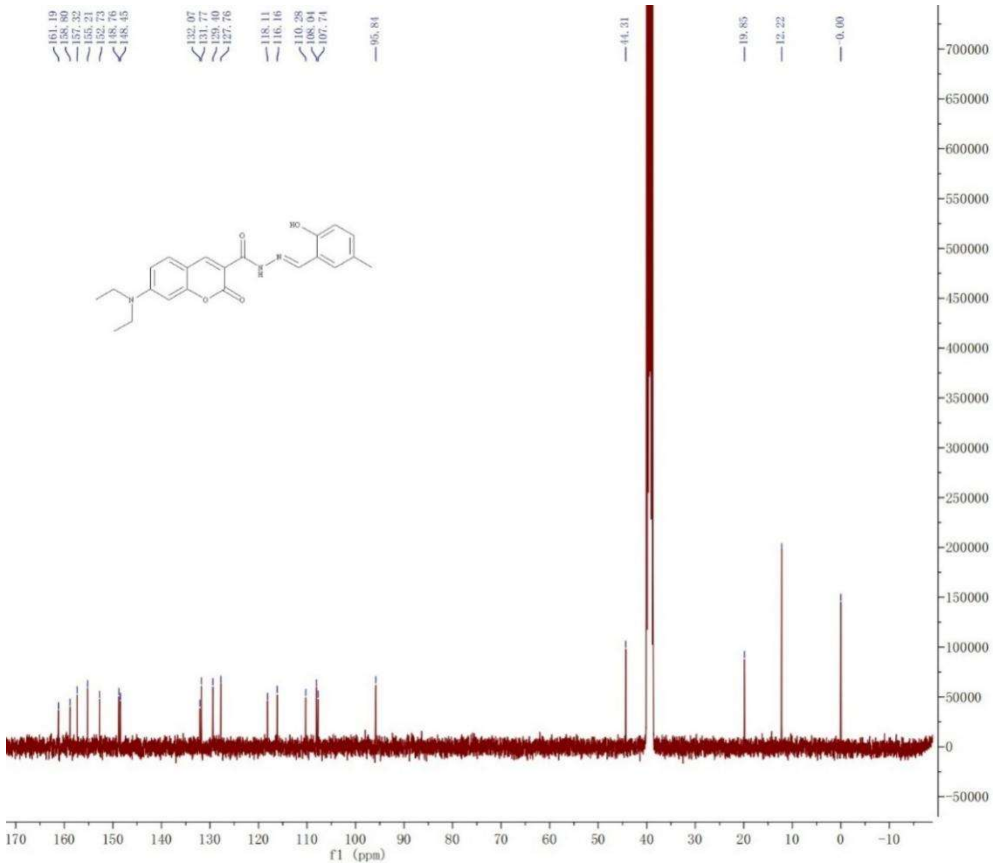

Figure S5 ^{13}C-NMR (100 MHz, $CDCl_3$) spectrum of compound **L**

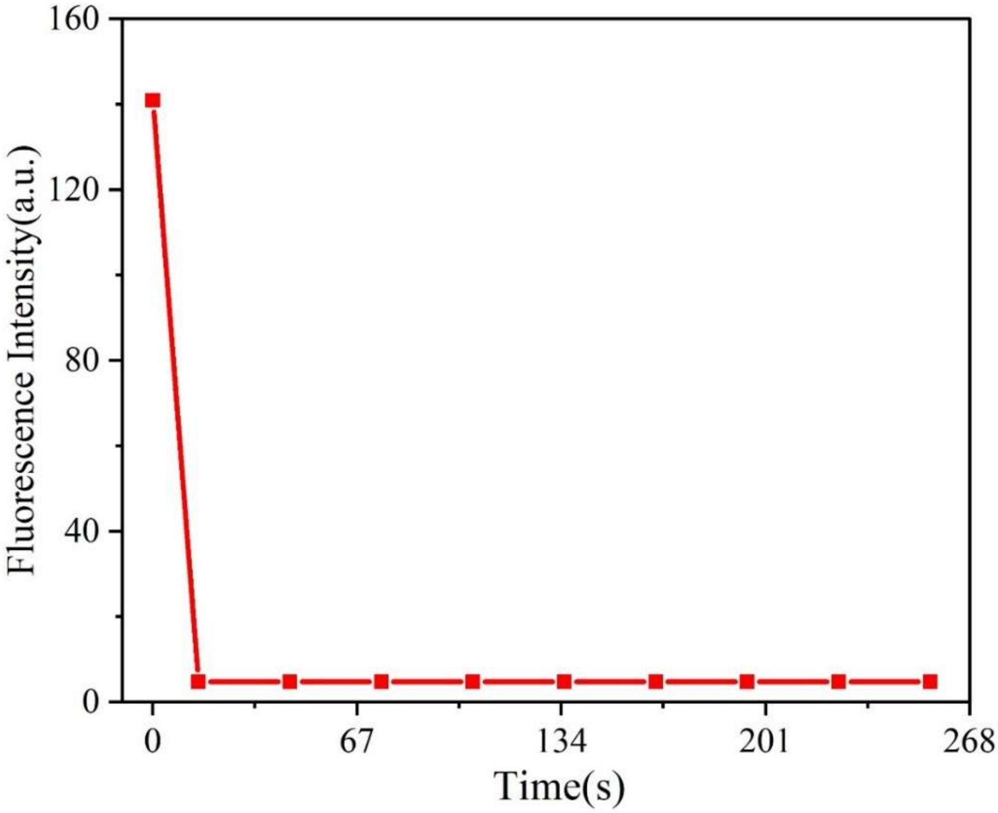

Figure S6 Fluorescence intensity variation with time after adding 3 equiv of Cu^{2+} in probe **L**

Calculation method of detection limit

The detection limit was calculated according to the formula LOD=3σ/S, where σ is the standard deviation of 20 blank samples, and S is the linear regression slope between the fluorescence intensity of probe **L** at λ=484 nm and the concentration of

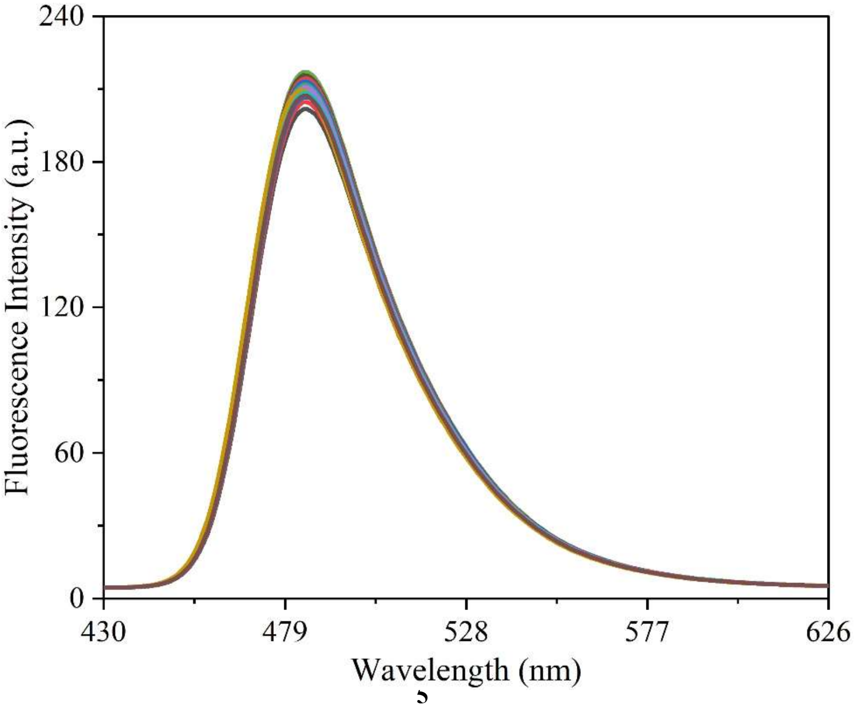

metal ions.

$$\sigma = \sqrt{\frac{1}{N-1}\sum_{i=1}^{N}(x_i - \overline{x})^2}$$

Figure S7 Fluorescence spectra of **L** without Cu^{2+} measured twenty times

Scheme S1 Synthesis of $Cu(ClO_4)_2L$

The square pyramid copper complex was readily prepared by layered diffusion method at room temperature. A clear ethanol solution (20 mL) of $Cu(ClO_4)_2 \cdot 6H_2O$ (37.05 mg, 0.1 mmol) was carefully layered onto a solution of **L** (39.34 mg, 0.1 mmol) in chloroform (20 mL). Brown blocky crystals suitable for X-ray diffraction were obtained by slow interlayer diffusion.

Table S2 Selected Bond Lengths (Å), Bond Angles (o) and Dihedral Angles of **L** and **$CuLClO_4$**

	**L**	**$CuLClO_4$**
Bond	lengths (Å) [a]	lengths (Å) [a]
C(11)-O(15) (L)/C(9)-O(2) (**$CuLClO_4$**)	1.2189(18)	1.263(3)
C(11)-N(12) (L)/C(9)-N(2) (**$CuLClO_4$**)	1.3607(19)	1.342(3)
N(12)-N(13) (L)/N(2)-N(1) (**$CuLClO_4$**)	1.3671(16)	1.379(3)
C(14)-N(13) (L)/C(8)-N(1) (**$CuLClO_4$**)	1.2807(18)	1.290(3)
C(14)-C(16) (L)/C(8)-C(6) (**$CuLClO_4$**)	1.4507(19)	1.435(3)
C(17)-O(22) (L)/C(1)-O(1) (**$CuLClO_4$**)	1.3592(17)	1.332(3)
Angles	(^{o})	(^{o})
C(11)-N(12)-N(13) (L)/C(9)-N(2)-N(1)	116.67(13)	114.37(19)
N(12)-N(13)-C(14) (L)/C(8)-N(1)-N(2)	120.38(14)	120.69(19)
Dihedral angle	(^{o})	(^{o})
P(C2-C11-N12)/P(N12-N13-C14) (L)/ P(C10-C9-N2)/P(N2-N1-C8)	3.06	6.32

Table S3 Comparison of representative Cu^{2+} fluorescent sensors with the present work

Sensors	LOD (μM)	Time (s)	$K_a(M^{-1})$	Methods for determination of binding constants	Ref.
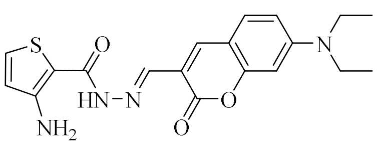	1.76×10^{-1}	30	1.19×10^{4}	ESI-MS, Job's plot	1
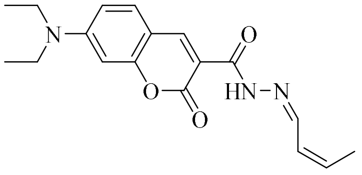	3.7×10^{-1}	N/A	N/A	Job's plot	2
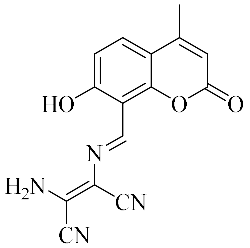	3.9×10^{-4}	30	2.37×10^{3}	ESI-MS	3
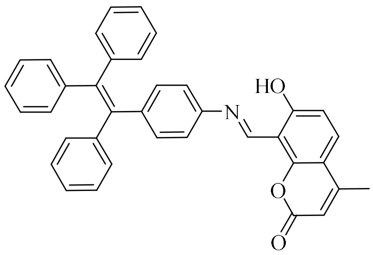	3.6×10^{-1}	immed iately	N/A	Fluorescence titration, ^{1}H-NMR	4
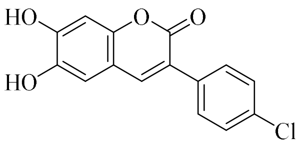	1.47×10^{-3}	60	1.23×10^{4}	ESI-MS, Job's plot	5
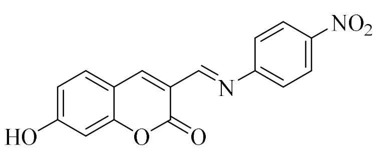	3.3×10^{-2}	35	6.9×10^{6}	Fluorescence titration, Job's plot	6
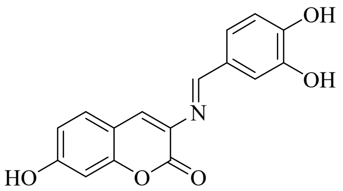	50	N/A	N/A	N/A	7

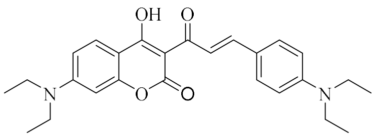	11.2	N/A	7.31×10^{3}	ESI-MS, Job's plot, Fluorescence titration	8
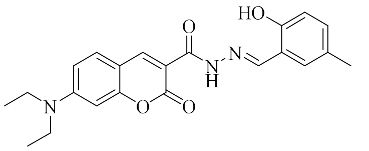	7.8×10^{-1}	15	5.2×10^{6}	Fluorescence titration, Job's plot, complex crystal	This work

1. Ma, Q.; Yang, X.; Zhao, Y. Development of a Coumarin-Based Schiff Base Fluorescent Probe and its Application in Detection of Cu^{2+}. *J.Fluoresc*. **2025**, *35*, 1-12.

2. Pang, S.; Yu, Y.; Yan, X.; Wu, M.; Liu, Q.; Zu, P.; Wu, C. Synthesis of Coumarinylhydrazone Fluorescent Probe and its Relay Recognition of Cu^{2+} and HPO_4^{2-}. *J. Fluoresc*. **2024**, *35*, 1-9.

3. Tang, Y.; Li, Y.; Han, J.; Mao, Y.; Ni, L.; Wang, Y. A coumarin based fluorescent probe for rapidly distinguishing of hypochlorite and copper (II) ion in organisms. *Spectrochim. Acta A*. **2018**, *208*, 299-308.

4. Wang, Y.; Hao, X.; Liang, L.; Gao, L.; Ren, X.; Wu, Y.; Zhao, H. A coumarin-containing Schiff base fluorescent probe with AIE effect for the copper(II) ion. *RSC Adv*. **2020**, *10*, 6109-6113.

5. Tahir, S.; Abdurrahman, K.; Nihan, KEŞ.; Duygu, A.; Furkan, Ö.; Kenan, K.; Nur, AF.; Orhan, GA.; İbrahim, Y. Fluorescent sensing platform for low-cost detection of Cu^{2+} by coumarin derivative: DFT calculation and practical application in herbal and black tea samples. *Turk. J. Chem*. **2020**, *44*, 1148-1163.

6. Abbas, WR.; Kadhim, MA. Design and synthesis of new coumarin-based fluorescent chemosensors for the dual detection of Hg^{2+} and Cu^{2+} in aqueous and biological samples. *RSC Adv*. **2025**, *15*, 44611-44622.

7. García-Beltrán, O.; Cassels, B.; Pérez, C.; Mena, N.; Núñez, M.; Martínez, N.; Pavez, P.; Aliaga, M. Coumarin-Based Fluorescent Probes for Dual Recognition of Copper(II) and Iron(III) Ions and Their Application in Bio-Imaging. *Sensors* **2014**, *14*, 1358-1371.

8. Li, K.; Huang, Y.; Sun, Y.; Zhang, Y.; Zhang, Y.; Ren, B.; Cao, D. A hydroxyl coumarin-chalcone-based fluorescent probe for sensing copper ions in plant and living cells. *J. Photoch. Photobio.B*. **2025**, *270*, 113218.
